# Exploring the arcuate fasciculus from a clinical perspective

**DOI:** 10.3389/fnins.2023.1307834

**Published:** 2023-11-15

**Authors:** Zhi Ding Shao, Yu Juan Gong, Jing Ren, Ji Wang

**Affiliations:** Second Affiliated Hospital of Wannan Medical College, Wuhu, Anhui, China

**Keywords:** arcuate fasciculus, diffusion tensor imaging, stroke, language ability, brain tumor

## Abstract

In recent years, language function impairment caused by intracranial diseases has gained increasing interest, mainly due to its significant impact on the language and cognitive ability, leading to a serious decline in the quality of life of patients. Consequently, researchers aimed to clarify the quantitative degree of lesions of the arcuate fasciculus and therapeutic targets to promote nerve fiber remodeling. The arcuate fasciculus is extremely prone to damage caused by diseases such as stroke and brain tumor. Hallucinating schizophrenia, autism spectrum disorder, epilepsy, chronic fatigue syndrome, chronic tinnitus, and other diseases can also lead to changes in the fractional anisotropy value of arcuate fasciculus; however, different studies have different conclusions about how this change occurs. To obtain a better understanding, more clinical studies are required. Owing to various advancements in neuroimaging, a better understanding and identification of vital targets for restoration of neurological function are possible. The arcuate fasciculus is stratified into three substructures, each having unique neurological functions. Both diffusion tensor imaging (DTI) sequences and deterministic monitoring techniques render it possible to visually and quantitatively analyze the substructure in three parts. In this review, we examined the progress of the arcuate fasciculus and quantitative DTI technology in recent years.

## Introduction

Complex language expression is a neurological function unique to humans. From birth to death, practicing language has been a continuous process, and concurrently, the progress of language has continuously promoted the exchange of information between people. In recent years, brain structures related to language function have been found to be closely associated with cognitive function (Yang et al., 2017; Cheema and Cummine, 2018). Research on post-stroke aphasia suggests that language-related white matter fiber tract pathways, such as arcuate fasciculus (AF), may have decreased FA values, which are related to language receptive and expressive abilities and language repetitive function (Yang et al., 2017). In patients with cerebral hemorrhage and aphasia, the left arcuate fasciculus was found to have varying degrees of changes in imaging characteristic values, such as FA values; another study on aphasia after cerebral infarction also suggested that the left arcuate fasciculus had decreased FA values, which were related to aphasia function (Koyama and Domen, 2016). Language function loss caused by various brain diseases has been found to not only affect the quality of life of patients but also lead to abnormalities in certain neurocognitive functions (Catani and Mesulam, 2008). Patients who had undergone brain glioma surgery were found to have significantly enhanced visualization of the left arcuate fasciculus, which was associated with the recovery of the patients’ language function (Hayashi et al., 2012); another similar study pointed out that the FA of the arcuate fasciculus in the dominant hemisphere of patients with brain tumors before surgery was elevated and predicted recovery of language function in patients after surgery (Kinoshita et al., 2014). To identify targets that can restore language ability or to use more precise methods for the reversal of an abnormal brain structure, over the past few years, many language scientists have discovered a remarkable correlation between brain regions and language cognition; examined the correlation among various neurons in the cortex, different neurotransmitters, and language; and finally determined the physical interconnectivities. Language functionality here mainly refers to the neural function produced by white matter fibers connecting different brain areas. The arcuate tract is one of the most well-known white matter fiber tracts. We believe that a broader understanding of this brain fiber structure can provide novel insights into clinical diagnosis and treatment (Breier et al., 2011; Raffa et al., 2017; Chawla et al., 2019).

## Review of simple neuroanatomy theory

In the human brain, the arcuate tract is an important nerve fiber bundle, which is closely related to the human language function and is the main contact fiber of the language dorsal pathway (Bernal and Ardila, 2009). The AF is believed to link the Broca’s area within the dominant inferior frontal gyrus with the posterior Wernicke area to the dominant superior temporal gyrus (Geschwind, 1970). Anatomically, it is located lateral to the corticospinal tract, adjacent to the corpus callosum. The arcuate tract connects the prefrontal cortex and the posterior superior temporal gyrus on the dorsal side. The segment bends and enters the posterior temporal cortex (Catani and Mesulam, 2008; Axer et al., 2013). The AF is known to originate from Brodmann’s areas (BA) 22, 21, and 37 and terminates in BA 44, 45, and 46 (Hong et al., 2009; Jehna et al., 2017). Currently, most literature divide it into three parts: deep, anterior, and posterior arcuate fasciculus (Martino et al., 2013; Falkenberg et al., 2020). Owing to the advancement in scanning techniques and post-processing methods, more nuanced anatomical forms can be identified from noninvasive diffusion tensor imaging (DTI) methods (Bernard et al., 2019). Based on the above anatomy, several studies based on EEG and MRI have found that when patients have language dysfunction, they are often accompanied by varying degrees of damage to the arcuate fasciculus, and the location of damage is related to the type and degree of aphasia (Zhang et al., 2010), which can be understood as differences in neural functions of substructures (Janssen et al., 2023). It was also found that the left and right arcuate tracts exert different roles in language function (di Cristofori et al., 2021; Gao et al., 2022), which is closely related to neural function formation in ontogeny. However, if the findings of previous studies are also evaluated using more neurological diseases, people may have a more varied and new understanding of the anatomical structure and function of the arcuate fasciculus (di Cristofori et al., 2021).

## Progress in the recognition of the arcuate fasciculus

**Figure 1:**
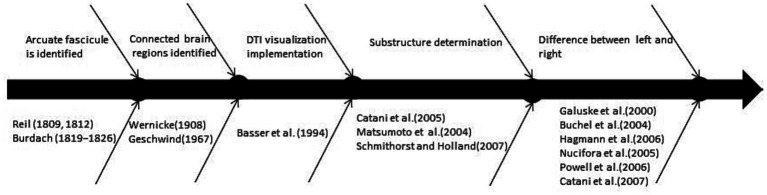
The timeline of the arcuate fasciculus is recognized by researchers.

The process of understanding the arcuate fasciculus is presented in Figure 1. First, confirm the existence of the arcuate fasciculus from the neuroanatomy, determine the brain regions connected by it, and then start exploring via noninvasive visualization to further understand a more detailed structural relationship, particularly, what are the major substructures. At this time, the completeness and quantitative accuracy of electroencephalogram (EEG), functional magnetic resonance imaging (fMRI), and DTI technologies were gradually attained. Furthermore, in combination with behavioral tests, it is found that in right-handed people, the left arcuate tract mainly participates in language function, whereas the right side participates in language adjustment. Thus, this verifies that both sides of the arcuate tract are involved in cognitive functions and that each part of the substructure has a different role in language participation, as evidenced from fMRI and EEG results, among others (Catani and Mesulam, 2008). Thus, understanding of the arcuate bundle is a constant process. With the ongoing technological advancements, more discoveries will be uncovered.

## Arcuate fasciculus and language

Traditionally, language is split into several subsystems as follows (van der Lely and Pinker, 2014): syntax, morphology, phonology, pragmatics, and lexicon. We have herein focused on the first three subsystems and proposed to cross-classify them via distinction of representation and processing. In the cranium, the brain structure related to it is described as follows: the main function of the dorsal pathway is a hierarchical phrase structure and syntactically complex sentences (extended syntax), rule-generated regular inflection (extended morphology), acoustic to articulatory network (sensorimotor interface), acoustic to articulatory network (speech segments; basic phonology), and acoustic to the articulatory network (syllables and larger prosodic domains; extended phonology); the main functions of the ventral and bilateral pathways are local phrase structure (basic syntax) and lexical and phrasal semantics (basic syntax), stored irregular forms (basic morphology), stored derived forms (basic morphology), and acoustic to semantic network (word recognition; basic phonology; van der Lely and Pinker, 2014). This article will not elaborate further on more biologically related issues of complex and delicate language as this review only aimed to illustrate the anatomy and function of the arcuate tract in combination with the clinical utility of advanced magnetic resonance sequences to help patients with language dysfunction. To realize the above functions, the coordination of different brain regions is required; thus, the connection between regions and neurotransmitter transmission depends on the white matter fiber structure. Certainly, current techniques still fail to clearly elucidate all brain structures related to language ability in the entire brain; however, the vital and main arcuate tracts have provided opportunities for research. A recent good study combined task fMRI and DTI to confirm the neural function of the substructure of the arcuate fasciculus in language execution and further differentiated the more accurate language-related tasks that different substructures are responsible for Janssen et al. (2023).

## Arcuate fasciculus and DTI

**Figure 2:**
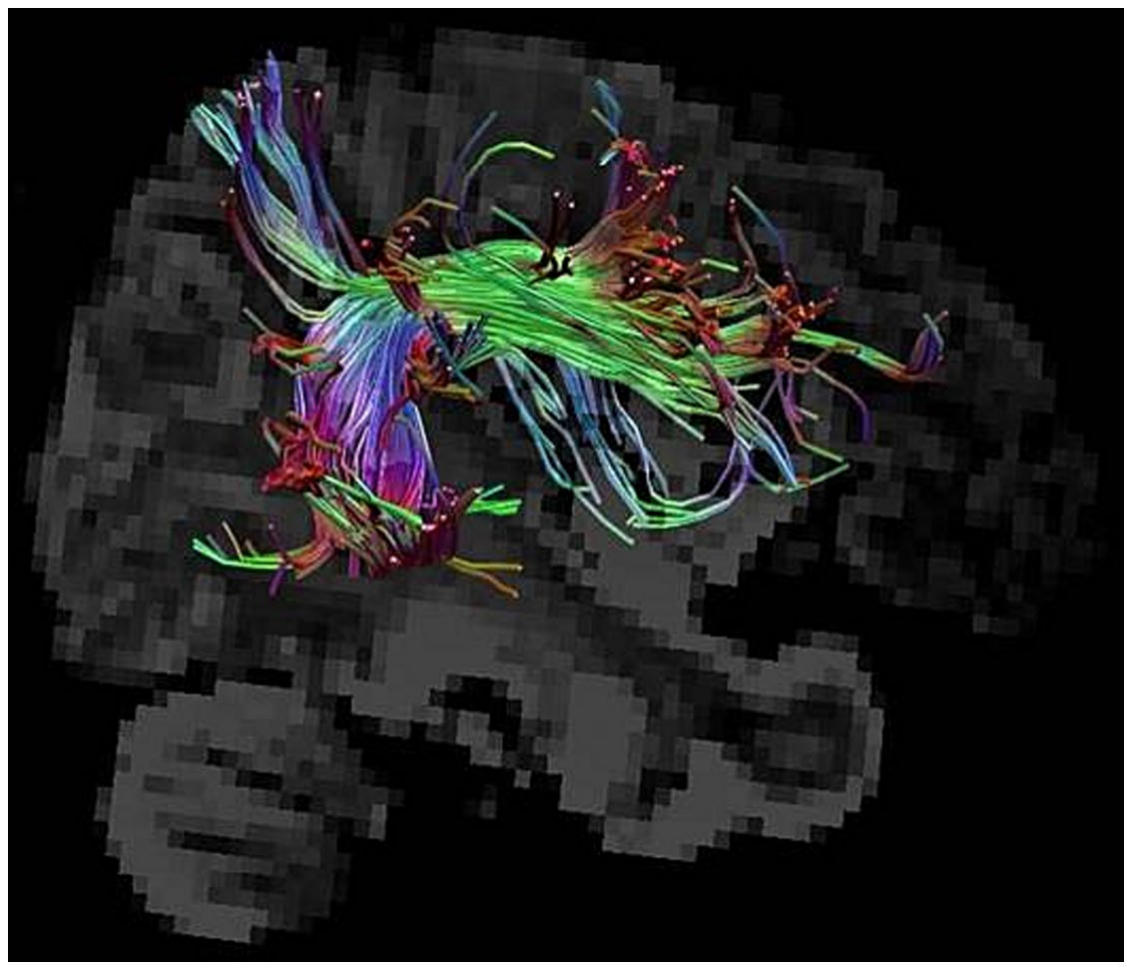
Visualization of the arcuate fasciculus by DTI.

From the end of the 19th century to the present, the relationship between brain white matter fiber structure and language has been perpetually explored, and the technological advancements are streamlined daily. Among these advancements, diffusion-weighted magnetic resonance imaging, also known as DTI, allowed tracking of white matter fiber structure *in vivo* (Janssen et al., 2023). The post-processing techniques of such sequence are mainly divided into deterministic and probabilistic fiber tracking. In the deterministic fiber tracking technology, through neuroanatomy, a variety of white matter fiber structures are confirmed; thus, it has a high degree of reliability and restoration authenticity. For example, the arcuate bundle, which is the highlight of this review, is difficult to display via conventional MRI techniques, let alone the substructure, while DTI sequences can provide very clear morphological structures such as that in Figure 2. After restoration of this brain structure noninvasively, as a clinician, a question is posed: how can it be used and researched? Thus far, we found that this technology can be utilized more in stroke and brain tumor. For brain tumors, it can be precisely positioned prior to surgery to significantly improve the quality of life of patients after surgery, and secondly, it can be used to predict neurological function recovery, such as language and cognition after surgery (Mori and van Zijl, 2002; Yang et al., 2017; Zimmermann et al., 2020). For stroke, the highlight is on predicting the recovery of language ability and exploring new methods of diagnosis and treatment, such as neuromodulation therapy (Puig et al., 2017; Wang et al., 2020)^.^

## Visualization of the arcuate fasciculus structure

**Figure 3:**
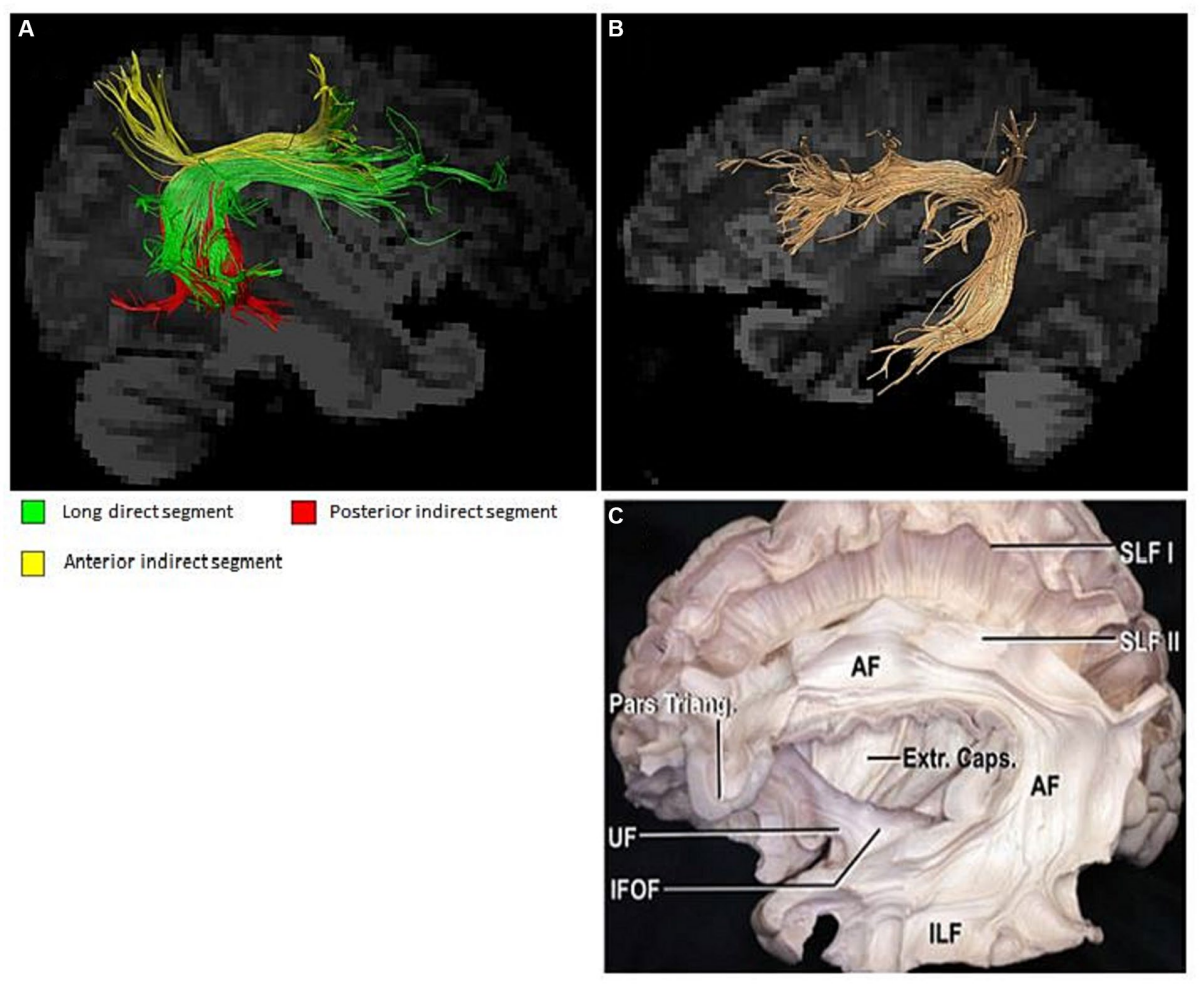
**(A)** Substructure of the arcuate fasciculus; **(B,C)** Arcuate fasciculus and neuroanatomy (Yagmurlu et al., 2016).

The visualized structure obtained by various post-processing methods allows an intuitive observation of morphological structure changes of a certain position of the arcuate tract. If it is determined that the changed structure is related to certain neural functions, the neural functions involved in the structure can be qualitatively determined. It is instrumental in helping patients better understand their own condition and can be used as a diagnostic marker for clinically investigating more treatment options (Yagmurlu et al., 2016). The current visualization of the arcuate tract is quite advanced and has been confirmed by neuroanatomists (Yagmurlu et al., 2016). This provides precise opportunities for conducting both cross-sectional and longitudinal studies (Figure 3). The presentation of its three-part substructure has also been realized, and many research groups have conducted studies on the correlation between the substructure and behavior and whether a relationship between the substructure and other brain regions exists or whether there is participation in the realization of certain neural functions. Reviewing past research data, varying scan parameter selections have different rendering quality for the three parts of the arcuate beam; therefore, a better quantification is also warranted. Regarding whether the identification is impaired, in the routine sequence, some studies have elucidated that even if no lesion is found on DWI, DTT can certainly demonstrate the absence of the arcuate tract in patients, which corroborates with the responsible area of the patient’s language dysfunction. Yamada et al. reported a patient who showed conduction aphasia after corona infarction. The patient exhibited severe dysrepetition with speech errors. Although no lesions were observed in the Broca’s or Wernicke’s areas on brain diffusion-weighted images, DTT demonstrated partial damage to the left AF in terms of FA values and AF configuration: a decreased FA value and a smaller left AF size, compared with the right AF (Yamada et al., 2007). Another Korean research group revealed that patients with aphasia caused by cerebral hemorrhage presented with different types of aphasia under different AF structures, and visual AF allowed a clear observation of the morphological changes that generated obvious differences (Ryu and Park, 2020). These findings should fully attract the attention of clinicians. For patients who present with neurological deficits but with no abnormalities in routine examinations, improving more sequence examinations and conducting a follow-up analysis to provide an accurate diagnosis and prognosis and derive new treatment strategies are warranted.

## Indices predicting language function recovery

If visualization aimed to match the neuroanatomy and help in better understanding the brain structure, then the core should be in its quantifiable characteristics, that is, values can be quantitatively identified under different degrees of damage to accurately predict the long-term prognosis of individuals. To predict the degree of language impairment recovery, the arcuate tract is not solely involved. If accuracy and precision are pursued, the nerve fiber structure near or distant from the lesion should also be included. The predicted value mainly comprised various types of eigenvalues from DTI sequences, such as FA, ADC, volume, and length values, among others (Wu et al., 2022). In recent years, the connection between post-stroke aphasia and arcuate tract changes has become one of the hotspots. Definitely, other different fiber tract pathways related to human language remain unexplored, and this review only elucidates on the arcuate tract. The more common arcuate tract injuries are still brain tumors and strokes, and the different degrees of damage can also predict whether the patient’s language ability can be restored in a few months. For example, left arcuate fasciculus changes have been detected among right-handed patients after stroke. Some studies have discovered that approximately 24%–38% of stroke patients may develop aphasia in the acute phase (Laska et al., 2001; Engelter et al., 2006). However, in patients with aphasia, lesions could not be detected in conventional MRI scan sequences. Therefore, perfecting DTI sequences can help in accurately classifying the types of aphasia (Kim et al., 2011), which will bring opportunities for targeted diagnosis and treatment. Another research group compared the intracranial DTI of the arcuate tract of aphasia in the acute phase of stroke with the control group and found a significant decline in the FA value of the left arcuate tract, thus confirming that the left arcuate tract is responsible for aphasia in post-stroke patients (Koyama and Domen, 2016). In the study of brain tumors inducing arcuate tract damage, AF quantification provided relevant guidance preoperatively (Hayashi et al., 2012). Studies have demonstrated that the preoperative increase in the FA value of the arcuate tract in the dominant hemisphere can be used as a predictor of language recovery after tumor resection, that is, the increase in the preoperative FA value of the arcuate tract suggests a high possibility for postoperative language recovery, and thus, surgery should be exercised with extreme caution to avoid unnecessary injury (Kinoshita et al., 2014). Another study further demonstrated that the change of the characteristic value of the arcuate tract under DTI sequence may be correlated with malignancy of a tumor, which indicates that noninvasive methods may help in determining the malignancy of the patient’s lesion and guide treatment (Zoli et al., 2021). In a research group in China using the DTT method to predict postoperative language function in low-grade gliomas, the volume and number of the arcuate tract fibers can afford better reliability than the FA value, which once again confirms the noninvasive archetype. Also, the description of the eigenvalues of the bundle has predictability (Wu et al., 2022).

## Therapeutic targets, precise targeting, and individualized treatment

Damage to different brain regions may have varied treatment options, such as the treatment site and transcranial magnetic stimulation intensity, and different neural regulation methods. A study differentiated the structure of white matter fibers related to exercise under TMS treatment and found that DTI-based guided neural regulation research is feasible (Raffa et al., 2018). Professors from Harvard Medical School and Boston University performed a very systematic study on the relationship between TMS treatment and brain white matter fiber tracts, and the study findings showed that DTI can be used to describe the microstructural description of TMS and the connection abnormalities of the different brain regions (Naeser et al., 2010). More studies have revealed that the arcuate tract is also related to the cognitive function of patients, and it is more manifested on both sides, rather than the left side. Thus, to improve cognitive symptoms, treatment strategies employed for arcuate tract changes through neurological regulatory methods are worthy of attention. For patients with brain tumors, it is of crucial importance to determine the extent of tumor resection prior to surgery and to predict the degree of postoperative speech function recovery. Studies have demonstrated that the combination of DTI and nTMS is the most optimal preoperative strategy, and patients enrolled in this method showed better prognosis and exhibited reduction in movement and language damage secondary to surgery (Raffa et al., 2017). Another study emphasized that DTI is effective and feasible in awake surgery as the arcuate bundle is a common and easily damaged structure during surgery, and the current visualization technology is advanced. Its appropriate application during surgery can help in deterring nerve function damage, such as those related to speech and movement (D'Andrea et al., 2016). Many studies have been published for the treatment of post-stroke aphasia with TMS. Although the treatment site, time, and type of aphasia are varied, majority of the studies have achieved improvement in language function outcomes (Arheix-Parras et al., 2021). These researchers also acknowledged that a mechanism to explain these improvements is lacking. However, it is conjectured that the structural changes of the arcuate fasciculus may be the most intuitive factor (Arheix-Parras et al., 2021). Moreover, the relationship between the specific substructure changes of the arcuate fasciculus and TMS should be examined more to further refine diagnosis and treatment.

## Observing changes in neuroplasticity

One of the foundations of neurological recovery is neuroplasticity. For example, to evaluate the treatment outcomes, remodeling of nerve fibers at the damaged site should be observed. Evidence reveals that neural remodeling continuously occurs throughout a person’s lifetime (Kong et al., 2016). Even with disease conditions, the brain tissue itself has a certain capacity to remodel. Therefore, the occurrence of neural remodeling indicates restoration of lost neural function. Furthermore, if this mechanism can be elucidated more clearly, the symptoms can be improved and the quality of life of affected patients can be enhanced by targeting the formation of nerve remodeling after the occurrence of various intracranial diseases. Studies have confirmed that in the magnetic resonance imaging and MEG detection of patients with brain tumors, additional activated sites can be found, which can be verified as a form of neural remodeling combined with behavioral studies (Breier et al., 2011). Moreover, this can further clarify the mechanism or the relevant brain region connection via further imaging and white matter fiber structure reconstruction. A research group administered a standardized treatment on a patient with post-stroke aphasia and concurrently completed behavioral and DTI examinations in the acute phase and 3 months after the stroke. Improvements were observed on both time periods, accompanied by the improvement of the patient’s speech ability (Kierońska et al., 2021). Studies have also explored on post-stroke aphasia using various imaging modalities such as DTI, fMRI, and EEG and found changes in the brain structure or brain function related to language after 3 months of systemic treatment. The arcuate tract showed an increase in integrity and increase in the number and volume of fibers which directly correlate with recovery of language and cognitive functions (Breier et al., 2011). Another group of researchers also compared the structure of deep brain fiber tracts of gymnasts and non-athletes, in which the arcuate tract was found to have a regionally lower FA, which indirectly indicated that the arcuate tract may also be involved in motor regulation and spatial attention. The author speculates that the regular quantitative changes are associated with the time of exercise training received, and the researchers also used these quantitative fiber structure values to verify whether they can distinguish between athletes and non-exercise abilities (Deng et al., 2017).

## Discussion

Research on neuropsychological diseases using AF and/or in combination with other adjacent fiber tracts, such as diagnostic markers, has also been conducted (Chawla et al., 2019). For example, in hallucinating patients with schizophrenia, the left AF is longer, indicating increased connectivity between auditory areas in the temporal lobe and frontal and parietal areas associated with language (Fernández-Miranda et al., 2015). In bilateral AF, auditory hallucinations had significantly lower FA values (Chawla et al., 2019). Studies have found that toddlers with autism spectrum disorder (ASD) and left-sided AF have FA values significantly lower than those of normal individuals, suggesting the lack of speaking ability in the early stages of patients with ASD (Zhang et al., 2018). Clinically, it is common to observe personality changes in patients with epilepsy. A previous study reported that the intracranial white matter fiber tracts in patients with epilepsy undergo changes in multiple brain regions, thereby explaining the causes of personality changes. This study found that AF did not differ between patients with epilepsy diagnosed 5 years ago and normal controls, but AF changed significantly in patients diagnosed with epilepsy 12 years ago (Mao et al., 2011). Recent studies have shown that the right arcuate fasciculus undergoes changes in patients with chronic fatigue syndrome (Zeineh et al., 2015). In right-handed patients, the FA value of the right arcuate fascicle is significantly increased. According to the authors of this study, CFS is the cause of this change, and this change may serve as a diagnostic biomarker (Zeineh et al., 2015). It was also surprising to find a correlation between behavioral scores and changes in the left arcuate fasciculus in patients with tinnitus (Ahmed et al., 2021). Thus, from the above researches, we can conclude that with the advancement of neuroimaging and anatomical techniques, a better understanding of the arcuate tract is also achieved. There are many cells involved in the damage and remodeling of nerve fiber bundles, and the relationship between these biological changes and imaging findings needs to be clarified. Thus, a more detailed and complete research on the arcuate tract in relation to clinical diagnosis and treatment is warranted, and the conclusion from multicenter big data is an exciting pursuit.

## Author contributions

ZS: Resources, Writing – review & editing. YG: Methodology, Writing – review & editing. JR: Formal analysis, Writing – review & editing. JW: Software, Writing – original draft, Writing – review & editing.
