# Supplementary figures and images for: Exploring the arcuate fasciculus from a clinical perspective
Source: Front Neurosci. 2023 Nov 15;17:1307834. doi: 10.3389/fnins.2023.1307834 (PMC10684764; doi:10.3389/fnins.2023.1307834)

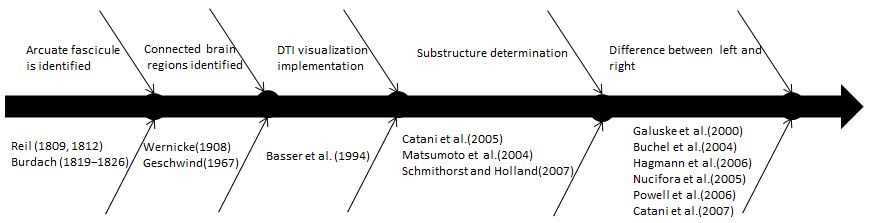

Supplement: Supplementary file 1 [file Image_1.JPEG]
